# Modelled impact of virtual fractional flow reserve in patients undergoing coronary angiography (VIRTU-4)

**DOI:** 10.1136/heartjnl-2024-324039

**Published:** 2024-05-16

**Authors:** Mina Ghobrial, Hazel Haley, Rebecca Gosling, Daniel James Taylor, James Richardson, Kenneth Morgan, David Barmby, Javaid Iqbal, Arvindra Krishnamurthy, Rajender Singh, Dwayne Conway, Ian Hall, Zulfiquar Adam, Nigel Wheeldon, Ever D Grech, Robert F Storey, Alexander Rothman, Gillian Payne, Muhammad Naeem Tahir, Simon Smith, Justin Cooke, Steven Hunter, Neil Cartwright, Syed Sadeque, Norman Paul Briffa, Abdallah Al-Mohammad, Laurence O’Toole, Dominic Rogers, Patricia V Lawford, David R Hose, Julian Gunn, Paul D Morris

**Affiliations:** 1 Division of Clinical Medicine, School of Medicine & Population Health, University of Sheffield, Sheffield, UK; 2 South Yorkshire Cardiothoracic Centre, Sheffield Teaching Hospitals NHS Foundation Trust, Sheffield, UK; 3 Insigneo Institute of In Silico medicine, University of Sheffield, Sheffield, UK; 4 NIHR Sheffield Biomedical Research Centre, Sheffield Teaching Hospitals NHS Foundation Trust, Sheffield, UK; 5 Doncaster and Bassetlaw Teaching Hospitals NHS Foundation Trust, Doncaster, UK; 6 Barnsley Hospital NHS Foundation Trust, Barnsley, UK; 7 The Rotherham NHS Foundation Trust, Rotherham, UK; 8 Chesterfield Royal Hospital NHS Foundation Trust, Chesterfield, UK

**Keywords:** Coronary Angiography, Coronary Artery Disease, Computer Simulation

## Abstract

**Background:**

The practical application of ‘virtual’ (computed) fractional flow reserve (vFFR) based on invasive coronary angiogram (ICA) images is unknown. The objective of this cohort study was to investigate the potential of vFFR to guide the management of unselected patients undergoing ICA. The hypothesis was that it changes management in >10% of cases.

**Methods:**

vFFR was computed using the Sheffield VIRTUheart system, at five hospitals in the North of England, on ‘all-comers’ undergoing ICA for non-ST-elevation myocardial infarction acute coronary syndrome (ACS) and chronic coronary syndrome (CCS). The cardiologists’ management plan (optimal medical therapy, percutaneous coronary intervention (PCI), coronary artery bypass surgery or ‘more information required’) and confidence level were recorded after ICA, and again after vFFR disclosure.

**Results:**

517 patients were screened; 320 were recruited: 208 with ACS and 112 with CCS. The median vFFR was 0.82 (0.70–0.91). vFFR disclosure did not change the mean number of significantly stenosed vessels per patient (1.16 (±0.96) visually and 1.18 (±0.92) with vFFR (p=0.79)). A change in intended management following vFFR disclosure occurred in 22% of all patients; in the ACS cohort, there was a 62% increase in the number planned for medical management, and in the CCS cohort, there was a 31% increase in the number planned for PCI. In all patients, vFFR disclosure increased physician confidence from 8 of 10 (7.33–9) to 9 of 10 (8–10) (p<0.001).

**Conclusion:**

The addition of vFFR to ICA changed intended management strategy in 22% of patients, provided a detailed and specific ‘all-in-one’ anatomical and physiological assessment of coronary artery disease, and was accompanied by augmentation of the operator’s confidence in the treatment strategy.

**WHAT IS ALREADY KNOWN ON THIS TOPIC**

- Using coronary physiology to guide intervention is superior to using angiography in rationalising interventions and improving clinical outcomes and health economics. Image-derived fractional flow reserve (FFR) based on invasive coronary angiography (ICA) is now entering clinical practice. It relies upon image-based arterial reconstructions and the application of the laws of fluid dynamics, adapted to the coronary circulation. This technology is starting to show benefit in controlled trials, but data are lacking in terms of ‘real-world’ applicability.

**WHAT THIS STUDY ADDS**

- Virtual FFR (vFFR) was computed in 320 ‘all-comers’ undergoing ICA for acute coronary syndrome or chronic coronary syndrome. As an adjunct to ICA, vFFR resulted in change in 22% of patients’ management plans.

**HOW THIS STUDY MIGHT AFFECT RESEARCH, PRACTICE OR POLICY**

- vFFR augments ICA by providing a real-time, ‘all-in one’ anatomical and physiological assessment of coronary artery disease. It is time for it to be more widely applied in routine practice to ensure more appropriate targeting of revascularisation. It also needs to be developed for use in wider patient groups such as in heart failure, arrhythmia and valve disease.

## Introduction

Invasive coronary angiography (ICA) is the final common pathway for patients with probable coronary artery disease (CAD) being considered for revascularisation. Traditionally, decision-making involves inspection of the ICA images and inferring the effect of any stenosis seen upon blood flow through the arterial lumen. This is both subjective and inaccurate,^1 2^ resulting in unnecessary procedures or undertreatment. Fractional flow reserve (FFR) or related indices can assess the ischaemic potential of a stenotic lesion^3^ and guide percutaneous coronary intervention (PCI), with benefits in terms of reduced adverse events^4–7^ and healthcare costs.^8^ FFR also changes management in both stable^9^ and unstable syndromes.^10^ Guidelines support the use of FFR and instantaneous wave-free ratio(iFR).^11 12^ However, logistic, practical and financial reasons prevent physiological guidance being widely employed in clinical practice.^13^ Therefore, several systems which compute ‘virtual’ FFR (vFFR) from ICA have been developed. The first of these was the VIRTUheart system (University of Sheffield, UK), employing computational fluid dynamic (CFD) modelling to calculate vFFR.^14^ Validation of vFFR against invasive FFR is acceptable,^15^ and data on clinical outcomes are emerging.^16 17^ However, their applicability and impact on decision-making in ‘real-world’ settings are lacking.

The VIRTU-4 Study aimed to investigate the impact on decision-making of vFFR in patients with acute coronary syndrome (ACS) or chronic coronary syndrome (CCS) undergoing ICA.

## Methods

### Setting

This was an investigator-initiated, multicentre, cohort, prospective observational study in which patients with ACS or CCS, who were scheduled to undergo ICA at a tertiary centre (patients with ACS) and four district hospitals (patients with CCS) in Northern England, were enrolled over the 2-year period (2020–2021).

### Patients and procedures

Patients were over the age of 18 years, presenting with CCS or ACS, requiring ICA, ±on-site invasive FFR assessment in the ACS group. In cases where FFR was measured invasively, this was done after intracoronary administration of nitrate, with a pressure-sensitive wire, with the transducer positioned at least 15 mm distal to the lesion, during maximal stable hyperaemia, induced by an intravenous infusion of adenosine at 140 µg/kg/min.^3^ Across the five hospital sites, 24 interventional cardiologists performed ICA. Exclusion criteria were serum creatinine >180 µmol/L, refractory ischaemia, haemodynamic instability, prior coronary artery bypass grafting (CABG), significant valvular disease, intolerance to antiplatelet drugs, life-threatening comorbidity and failure to consent. A second phase of exclusion, based on the angiographic requirements for modelling, included chronic total occlusion as the only lesion, left main stem or aorto-ostial disease (which is difficult to model), normal coronary arteries (<30% stenosis), lesions with >90% diameter stenosis (FFR being both unnecessary and difficult to model), vessel diameter <2.25 mm and inadequate images (eg, vessel overlap, inadequate contrast or inability to obtain two views). Angiographic images were acquired ensuring that each lesion was clearly displayed in at least two views, at least 30° apart. Angiographic disease was classified as non-significant (0VD) or one, two or three-vessel disease (1, 2 or 3VD), based on what the cardiologists believed to be potentially physiologically significant lesions (≥30% visual stenosis) by visual estimation as this most closely represented standard clinical practice. These were then reclassified following disclosure of vFFR, using a vFFR value of ≤0.80 as the threshold for functional significance. VIRTUheart was, therefore, deployed in all vessels with a visual stenosis of 30–90% as assessed visually by the research team (MG and HH) after ICA.

### Virtual fractional flow reserve

The VIRTUheart system that employs a three-dimensional (3D) quantitative coronary angiography segmentation is derived from two angiographic images, at end-diastole, displaying the lesion of interest. Arteries were reconstructed, by one of two operators, both experienced in the use of VIRTUheart and CFD modelling (MG or HH), from coronary ostium to at least six vessel radii distal to the lesion of interest. A CFD solver then resolves the Navier-Stokes and continuity equations, from which a pseudo-transient vFFR is calculated. The vFFR is superimposed in colour upon an image of the 3D anatomy.^14 18^ See central illustration for an example case of vFFR.

### Management strategy

The initial management strategy of the patient’s cardiologist was recorded after ICA, immediately after angiography, while the patient was on the table, before any PCI/FFR had been performed. This was categorised as optimal medical therapy (OMT), PCI, CABG or ‘more information required’, as this best reflects management categorisation of standard practice. The cardiologist was encouraged to commit to a strategy, formulated from the patient’s clinical history, comorbidities and angiographic images. The vFFR results were then disclosed to the cardiologist, and any changes in plan documented. Actual changes were not permitted because VIRTUheart is a research tool. The confidence level of the cardiologist in making their treatment decision was recorded at both stages.

### Endpoints

The primary endpoint was an intended change in management strategy after vFFR disclosure. This was defined as a change of plan between the categories of OMT, PCI, CABG or ‘more information required’ (invasive FFR, PCI plus invasive FFR or multidisciplinary team). Secondary endpoints included the number of vessels classified as significant, the confidence levels of the cardiologists, the vFFR failure rate, interobserver vFFR variability and agreement with invasively measured FFR (when measured).

### Sample size and statistics

Sample size was calculated based on the RIPCORD^9^ and FAMOUS-NSTEMI^10^ Studies, which showed that invasive FFR at the time of ICA changed management in >20% of patients. Assuming that vFFR might be less sensitive than measured FFR, we set our power level more stringently. 412 patients were required to provide 85% power at 5% significance to reject a change in treatment in <10% of patients. Categorical variables were presented as counts and percentages. Normally distributed data were presented as mean (±SD) or median (IQR), as appropriate. Normality of data distribution was assessed using the Shapiro-Wilk test. Primary and secondary outcomes were assessed using McNemar-Bowker, χ^2^, paired Student’s t-test and Mann-Whitney U tests, as appropriate. Differences in confidence levels before and after vFFR disclosure were assessed using one-way repeated measures analysis of variance. Interobserver variability between the two vFFR operators was assessed on randomly selected 10% of the patient cohort and compared using the intraclass correlation coefficient with a two-way mixed model. When comparing invasive and virtual FFR, correlation was assessed using Pearson’s correlation coefficient, agreement with Bland-Altman plots with associated 95% limits of agreement and diagnostic test performance with receiver operating characteristic (ROC) curve analysis.

### Patient and public involvement

The entire VIRTUheart Programme, which started in 2009, is reviewed each year by the Sheffield National Institute of Health Research Cardiovascular Patient Panel. For this study, they reviewed the protocol, provided advice concerning how to approach potential participants, and corrections for the patient information sheet and the consent form.

## Results

### Patients and vessels

**Figure 1:**
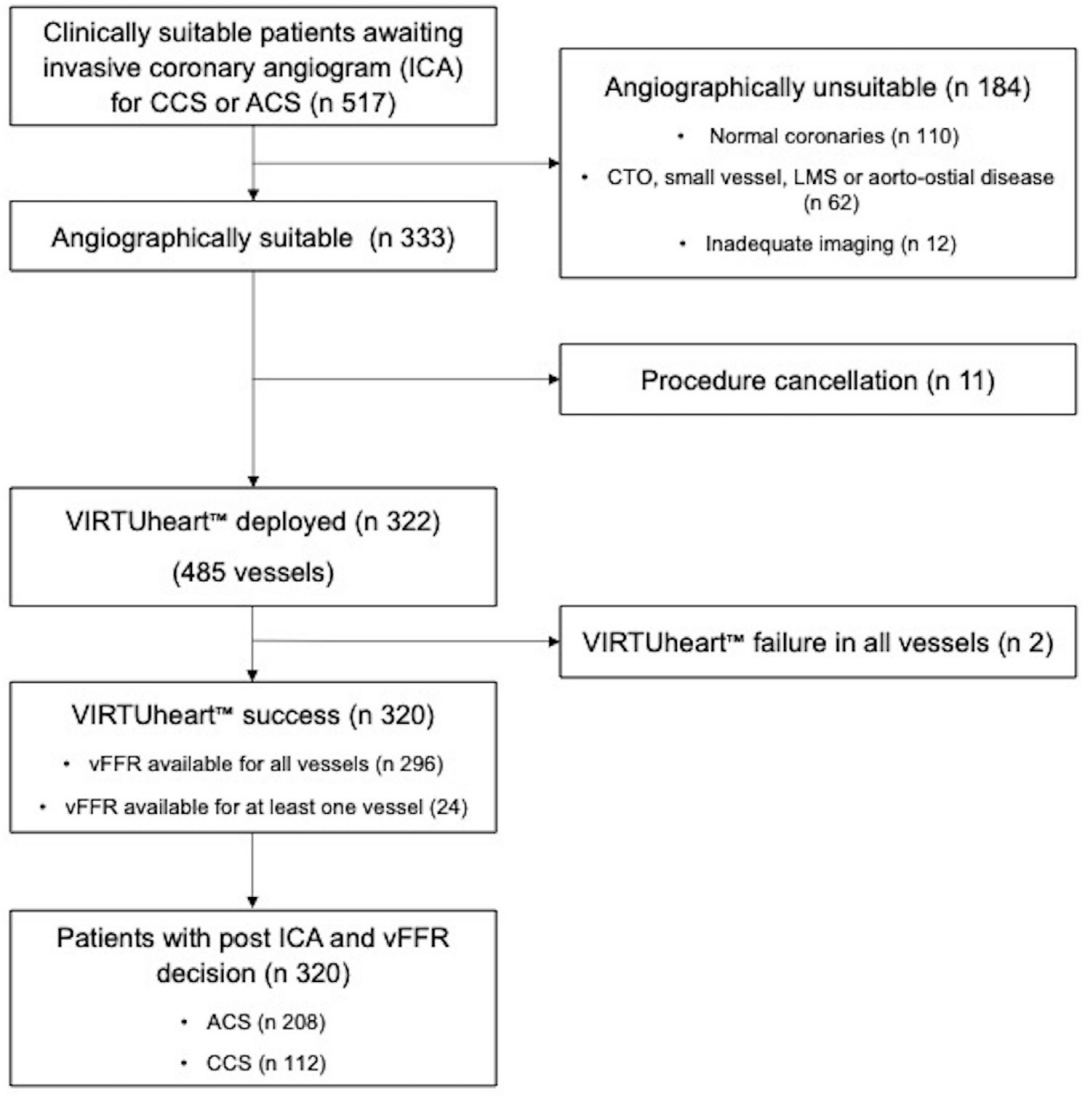
Consolidated Standards of Reporting Trials diagram. ACS, acute coronary syndrome; CCS, chronic coronary syndrome; CTO, chronic total occlusion; LMS, left main stem; vFFR, virtual fractional flow reserve.

Screening disclosed 517 clinically suitable patients between January 2020 and December 2021. After ICA, 320 of those were angiographically eligible for inclusion: 208 ACS and 112 CCS. Of the 197 patients excluded, 110 had normal coronary arteries, 62 had anatomical exclusions, 12 had inadequate imaging, 11 had procedure cancellations and the VIRTUheart system failed to converge to a stable solution in 2 cases. In a further 24, it failed in one, but not all, stenosed vessels, and these patients continued in the study. See figure 1 for the Consolidated Standards of Reporting Trials diagram, table 1 for demographics and table 2 for vFFR analyses.

**Table 1:** Baseline patient characteristics

Study characteristics	
**Total cohort, n (%)**	**320**
Age, mean (±SD)	65 (±11)
Male sex, n (%)	233 (73)
Diabetes mellitus, n (%)	94 (29)
Previous myocardial infarction, n (%)	59 (18)
History of treated hypertension, n (%)	188 (59)
History of treated hypercholesterolaemia, n (%)	190 (59)
History of smoking, n (%)	88 (28)
NYHA status, n (%)	
Class I	91 (28)
Class II	193 (60)
Class III	36 (11)
Frailty (Rockwood score) class classification, n (%)	
Well (1–3)	274 (86)
Vulnerable (4–5)	45 (14)
Frail (6–9)	1 (<1)
Echocardiography performed, n (%)	148 (46)
Primary/secondary preventative medications	
Antiplatelet, n (%)	314 (98)
Concomitant OAC, n (%)	22 (7)
Statin, n (%)	310 (97)
Mean serum creatinine, μmol/L	84 (±23)
**ACS cohort, n**	**208**
ECG evidence of ischaemia at presentation, n (%)	139 (67)
Peak troponin before the procedure	
Less ×5 upper limit of normal or 0, n (%)	27 (13)
More than ×5 upper limit of normal, n (%)	47 (23)
More than ×10 upper limit of normal, n (%)	134 (64)
GRACE score, median (IQR)	106 (90–126)
GRACE score >140, n (%)	20 (10)
Average time in days from index episode to catheterisation, mean (±SD)	5 (±2)
Radial access, n (%)	197 (95)
**CCS cohort, n**	**112**
Antianginals prescribed prior to angiography, n (%)	106 (95)
1 regular antianginal, n (%)	39 (35)
2 regular antianginals, n (%)	38 (34)
3 regular antianginals, n (%)	24 (22)
4 regular antianginals, n (%)	5 (4)
Non-invasive test performed prior to angiography, n (%)	66 (59)
SPECT, n (%)	35 (31)
CTCA, n (%)	17 (15)
ETT, n (%)	11 (10)
DSE, n (%)	2 (2)
pCMR, n (%)	1 (1)

ACS, acute coronary syndrome; CCS, chronic coronary syndrome; CTCA, CT coronary angiography; DSE, dobutamine stress echocardiogram; ETT, exercise tolerance test; GRACE, Global Registry of Acute Coronary Events; NYHA, New York Heart Association classification; OAC, oral anticoagulant; pCMR, perfusion cardiac magnetic resonance; SPECT, single-photon emission CT.

**Table 2:** Summary of vFFR analysis

Total vessels analysed	485
Left anterior descending artery, n (%) Left circumflex artery, n (%) Right coronary artery, n (%) Diagonal branch, n (%) Obtuse marginal branch, n (%) Posterior descending artery, n (%) Intermediate	208 (43) 82 (17) 111 (23) 37 (8) 25 (5) 13 (3) 5 (1)
Median vFFR	0.82 (0.70–0.90)
Median case calculation time (min)	16 (11–22.25)
VIRTUheart failure rate*, n (%)	26 (5.1)

*Technical failures.

vFFR, virtual fractional flow reserve.

### Extent and reclassification of CAD

**Figure 2:**
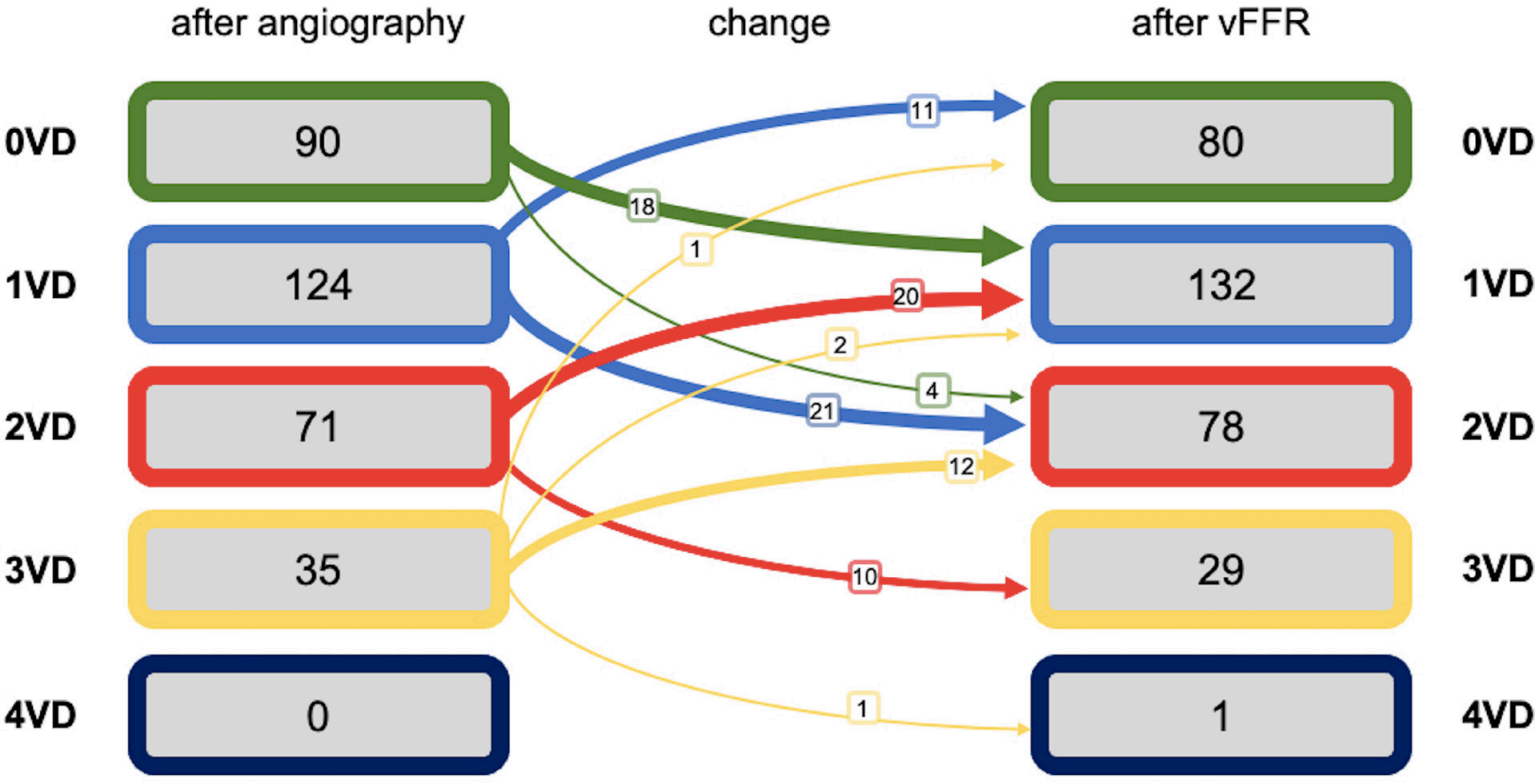
Classification of extent of significant disease before and after vFFR disclosure (N=320 patients). The widths of the arrows reflect the size of the effects. VD, vessel disease; vFFR, virtual fractional flow reserve.

The number of diseased arteries processed through VIRTUheart was 481 (table 2); the number of significant lesions per patient was 1.16 (±0.96) visually and 1.18 (±0.92) by vFFR (p=0.79) (see table 3 for individual patient reclassification). vFFR disclosure led to reclassification of 100 (31%) patients, 46 (14%) to fewer significantly diseased vessels (figure 2). The median vFFR was 0.82 (0.70–0.90). Table 4 shows the relationship between operator-assessed visual stenosis severity and vFFR-assessed lesion haemodynamic significance.

**Table 3:** Extent of disease by angiographic assessment and vFFR (McNemar-Bowker test, n=320 patients, p<0.001)

		Extent of significant disease according to vFFR				
		0-vessel disease	1-vessel disease	2-vessel disease	3-vessel disease	Totals
**Extent of angiographically significant disease**	**0-vessel disease**	41	6	0	0	47
	**1-vessel disease**	36	68	4	1	109
	**2-vessel disease**	11	43	41	1	96
	**3-vessel disease**	1	23	20	24	68
	**Totals**	89	140	65	26	320

vFFR, virtual fractional flow reserve.

**Table 4:** Stratified visually assessed lesion percentage stenosis and associated vFFR (χ^2^ p<0.0001)

Stenosis (%)	vFFR <0.8	vFFR ≥0.8	Total
30–50	3	58	61
51–70	32	83	115
71–90	123	36	159
Total	158	177	335

vFFR, virtual fractional flow reserve.

### Primary endpoint

#### Whole study population

**Figure 3:**
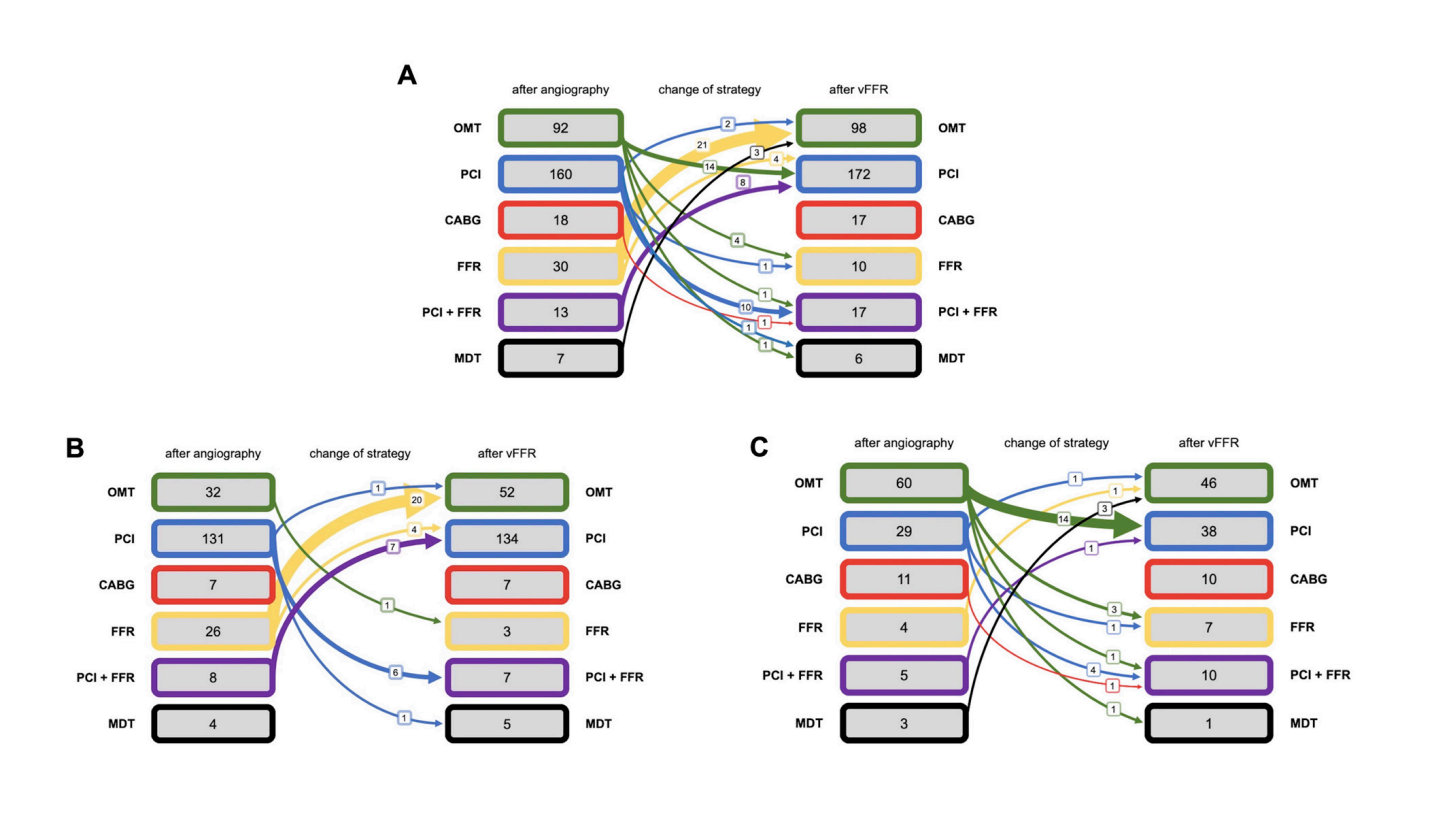
Details of management plans before and after vFFR disclosure. Only the changes are marked. In the remainder, vFFR did not alter the plan. (A) The entire cohort. The widths of the arrows reflect the size of the effects. (B) The patients with ACS. (C) The patients with CCS. ACS, acute coronary syndrome; CABG, coronary artery bypass grafting; CCS, chronic coronary syndrome; FFR, fractional flow reserve; MDT, multidisciplinary team; OMT, optimal medical therapy; PCI, percutaneous coronary intervention; vFFR, virtual FFR.

Management strategy changed in 71 of 320 (22%) patients after vFFR disclosure. The greatest single change was a decrease in the number of patients recommended to have wire-measured FFR, from 30 to 10. 25 switched out of the wire-measured FFR group and 5 switched in. 21 switched into the OMT group. 26 switched into the PCI group and 14 switched out, so the total changed only slightly, from 160 to 172. Despite the reclassification of individuals between treatment groups, the overall number in each group did not change significantly (p=0.25) (figure 3A).

#### The ACS group

Management strategy changed in 40 of 208 (19%) patients after vFFR disclosure. The greatest single change was a decrease in the number of patients recommended to have wire-measured FFR, from 26 to 3; 20 of the 23 switching to OMT. The number of patients recommended to have OMT increased from 32 to 52. 11 patients switched into the PCI group and 8 switched out, so the total remained almost constant (131 vs 134 after vFFR). In the ACS group, the reclassification of individuals between treatments groups significantly altered the proportion in each (p<0.01) (figure 3B).

#### The CCS group

Management strategy changed in 31 of 112 (28%) patients after vFFR disclosure. The greatest single change was a decrease in the number of patients recommended to have OMT, from 60 to 46, 14 switching to PCI. The number of patients recommended to have PCI or measured FFR increased from 33 to 45. In the CCS group, the reclassification of individuals between treatment groups did not significantly alter the proportion in each (p=0.08) (figure 3C).

#### Impact of vFFR on operator confidence

Median confidence scores in the treatment strategy before versus after vFFR disclosure were 8 (7–9) vs 9 (8–10), respectively (p<0.01). Confidence levels increased in the management of 165 (52%) patients, decreased in 30 (9%) and remained unchanged in 125 (39%).

#### Interobserver variability

Of the 41 random cases selected for interobserver variability testing, mean vFFR for operator 1 was 0.78 (±0.15) and for operator 2, 0.77 (±0.12). The correlation was 0.61 (p<0.01), the intraclass correlation coefficient was 0.75 (95% CI 0.53 to 0.87, p<0.01) and the degree of concordance for classifying each case as FFR >0.80 or <0.80 was 35 of 41 (85.4%). The coefficients of variation for operators 1 and 2 were 19% and 16% (p=0.41), respectively.

#### Comparison with invasive FFR

**Figure 4:**
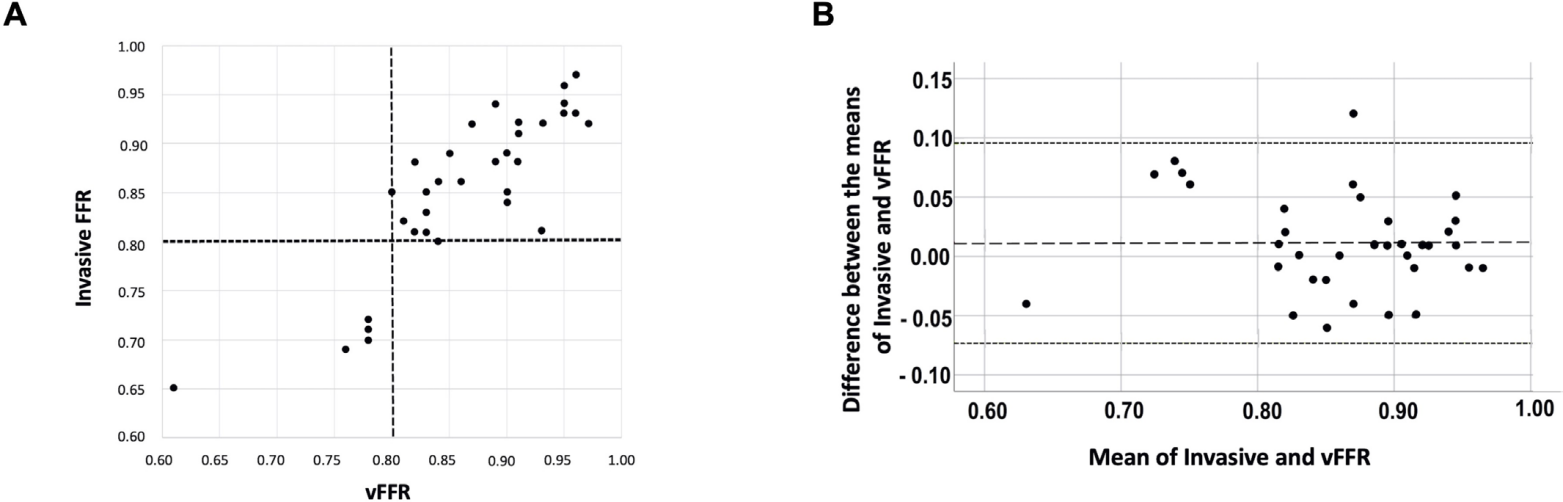
(A) Correlation between invasive FFR and vFFR. The broken lines indicate the ischaemic threshold of 0.80 (r=0.86, N=33). (B) Bland-Altman plot showing the relationship between each invasive and computed FFR. Overall bias (mean delta, broken line) was FFR +0.01 and limits of agreement (1.96 SD, dotted lines) were −0.07 to 0.10. FFR, fractional flow reserve; vFFR, virtual FFR.

33 (16%) patients with ACS underwent invasive FFR measurement for clinical reasons. The median invasive FFR was 0.87 (0.82–0.91) and vFFR 0.86 (0.81–0.92). The correlation and agreement between invasive FFR and vFFR was strong (r=0.86, p<0.01; bias +0.01, Bland-Altman limits of agreement ±0.08) (figure 4). For invasive assessment, six lesions met the threshold for haemodynamic significance (FFR ≤0.80). The diagnostic accuracy, sensitivity, specificity, positive and negative predictive values of vFFR compared with invasive FFR were 94% (95% CI 80% to 99%), 83% (95% CI 36% to 100%), 96% (95% CI 81% to 100%), 83% (95% CI 41% to 97%) and 96% (95% CI 81% to 99%), respectively. The area under the ROC curve was 0.95 (95% CI 0.85 to 1.00) indicating excellent discriminatory ability of vFFR compared with invasive FFR.

## Discussion

This study demonstrates that ICA-derived vFFR is applicable in 320 (62%) of 517 ‘all-comer’ patients undergoing clinically indicated ICA. Use of vFFR changed the management strategy in 71 (22%) patients. While there was some movement between groups, it did not alter the number of lesions deemed to be haemodynamically significant per patient (1.16 vs 1.18). In the ACS cohort, there was a 62% increase in the number proposed for OMT, whereas in the CCS cohort, there was a 27% decrease. In both groups, the use of vFFR overall increased the cardiologists’ confidence in their decision-making (central illustration).

The strength and originality of this study are its exploration of the ‘real-world’ impact of vFFR on everyday practice in both diagnostic and interventional cardiac catheter laboratories. The most important finding is the proportion of patients in whom it provided a change in management; 14% of those potentially suitable *before* the ICA was performed, and 22% of those in whom vFFR quantification was possible *after* ICA. In order to identify the 71 patients in whom vFFR would alter management, it was necessary to apply the software to 320. Nevertheless, screening five to benefit one seems a reasonable clinical yield. It is this ‘intermediate’ group, in which visual ambiguity around ICA interpretation exists, who may benefit from the addition of a physiological test. While invasive FFR is most widely used in clinical practice, application of vFFR may reduce procedural challenges, time and cost.

The overall proportion of patients in each category of management (OMT, PCI, CABG, ‘more information required’) before and after vFFR was similar for both all participants and the CCS subgroup, but there was significant crossover between treatment groups, so this may therefore represent more appropriate treatment allocation (figure 3), allowing for better targeting of effort and expenditure to achieve the best possible results in all patients. This is in accord with previous studies of measured^4–7 9^ and computationally derived^16^ FFR, the latter of which showed vFFR changed management decisions in 23% of patients with epicardial disease and is very similar to our finding of an overall change in management for 22% of patients.

The implications of vFFR in the subgroups diverged. In the ACS cohort, vFFR resulted in an 88% relative (6% absolute) reduction in the intended use of invasive FFR and a 63% relative (10% absolute) increase in the proportion assigned to OMT. The mean invasive FFR in this cohort was 0.86, perhaps reflecting the intended use of FFR as a tool to safely defer PCI in those considered for revascularisation.^4^ These findings complement those of RIPCORD 2, which showed that routine use of invasive FFR at the diagnostic stage (ICA only) is without benefit in selected patients with CCS and ACS.^19^ Unlike in RIPCORD 2, in which a pressure wire was deployed in all major epicardial vessels (median four), regardless of the presence of disease, in our study, vFFR was only deployed in lesions of uncertain severity, which accords better with normal practice. Therefore, vFFR, which is comparatively inexpensive on a patient-by-patient basis, and devoid of complications, could reap the benefits seen in previous, wire-measured FFR studies.

Somewhat different findings emerged in the CCS cohort. This group underwent cardiac catheterisation in non-PCI hospitals, where vFFR resulted in a 31% relative (8% absolute) increase in the proportion of patients allocated to PCI, despite an overall reduction in the extent of significant CAD. This finding was predominantly a consequence of 23% of medically managed patients being reallocated to single-vessel PCI after vFFR disclosure, 50% of whom had prior evidence of ischaemia on non-invasive testing—the extra evidence of localised ischaemia perhaps tipping the balance in favour of revascularisation in patients with CCS, despite current trends.^20–22^ Furthermore, in the CCS cohort, there was a 75% relative increase in the rate of invasive FFR referral after vFFR disclosure. Although the planned rate of invasive FFR use was lower in the CCS cohort compared with the ACS cohort, deploying vFFR in this group (in a non-PCI setting) could avoid a second procedure and attendant delays.^23^ This underlines the importance of education about the use of FFR and the ‘zone of uncertainty’ among non-interventional cardiologists to allow for cost-effective gate-keeping.^24^

The practicalities of using vFFR were encouraging. It was applicable in ‘real time’ in diverse cardiac catheterisation laboratories and allowed decisions to be made on the day of the procedure, which is an advantage for both the patient and cardiologist. Once deployed, the vFFR failure rate was low (3%) although, at the second screening stage, many ICAs did not provide the necessary lesion clarity in two views in diastole. vFFR was well received by consultants, and this translated into a small but significant increase in confidence in clinical decision-making. It was noticeable that confidence increased in cases in which clinical opinion accorded with the vFFR, and decreased when it differed. Validation of vFFR against invasive FFR values in the ACS cohort confirmed excellent diagnostic accuracy (94%).

### Limitations

First, this was a virtual trial, with changes in intended rather than actual management, and this may have affected the readiness of the cardiologists to propose changes. Second, the design did not provide an opportunity to assess actual clinical outcomes, because VIRTUheart is not clinically approved. Third, there were fewer patients with CCS than ACS, but that accurately reflects current clinical practice. Fourth, the study excluded patients with prior CABG, aorto-ostial and left main stem lesions, severe stenoses and diffuse CAD, all of which are regularly encountered. Fifth, vFFR makes assumptions about microvascular resistance, which can affect accuracy, unlike invasive FFR.^25 26^ Sixth, the cardiologists caring for the patients with ACS were interventionists and those caring for the patients with CCS were non-interventionists, so handling of the information may have differed. This, however, also reflects clinical pathways in many centres. Seventh, despite the consistency and high success rate of the two experts, their assessment of physiological significance differed in 15% cases, reinforcing doubts about the robustness and repeatability of this technology, especially if used by non-experts. Selection of angiographic views, timing of the selected frames, correction of the arterial outline and setting of the proximal and distal boundaries may all have contributed to this finding.^27^ Despite this, accuracy of the VIRTUheart system, which personalises microvascular resistance to patient-specific parameters,^28^ compared well with other systems.^15 25^

## Conclusion

ICA supplemented by vFFR has the potential to significantly change management strategies in approximately 22% of consecutive, real-world patients, providing a detailed and specific ‘one-stop-shop’ anatomical and physiological assessment of coronary disease, with appropriate treatment targeting and improved operator confidence.

10.1136/heartjnl-2024-324039.supp1 Supplementary data

## Data Availability

Data are available upon reasonable request.
