# Supplementary figures and images for: Modelled impact of virtual fractional flow reserve in patients undergoing coronary angiography (VIRTU-4)
Source: Heart. 2024 May 16;110(16):1048–55. doi: 10.1136/heartjnl-2024-324039 (PMC11287621; doi:10.1136/heartjnl-2024-324039)

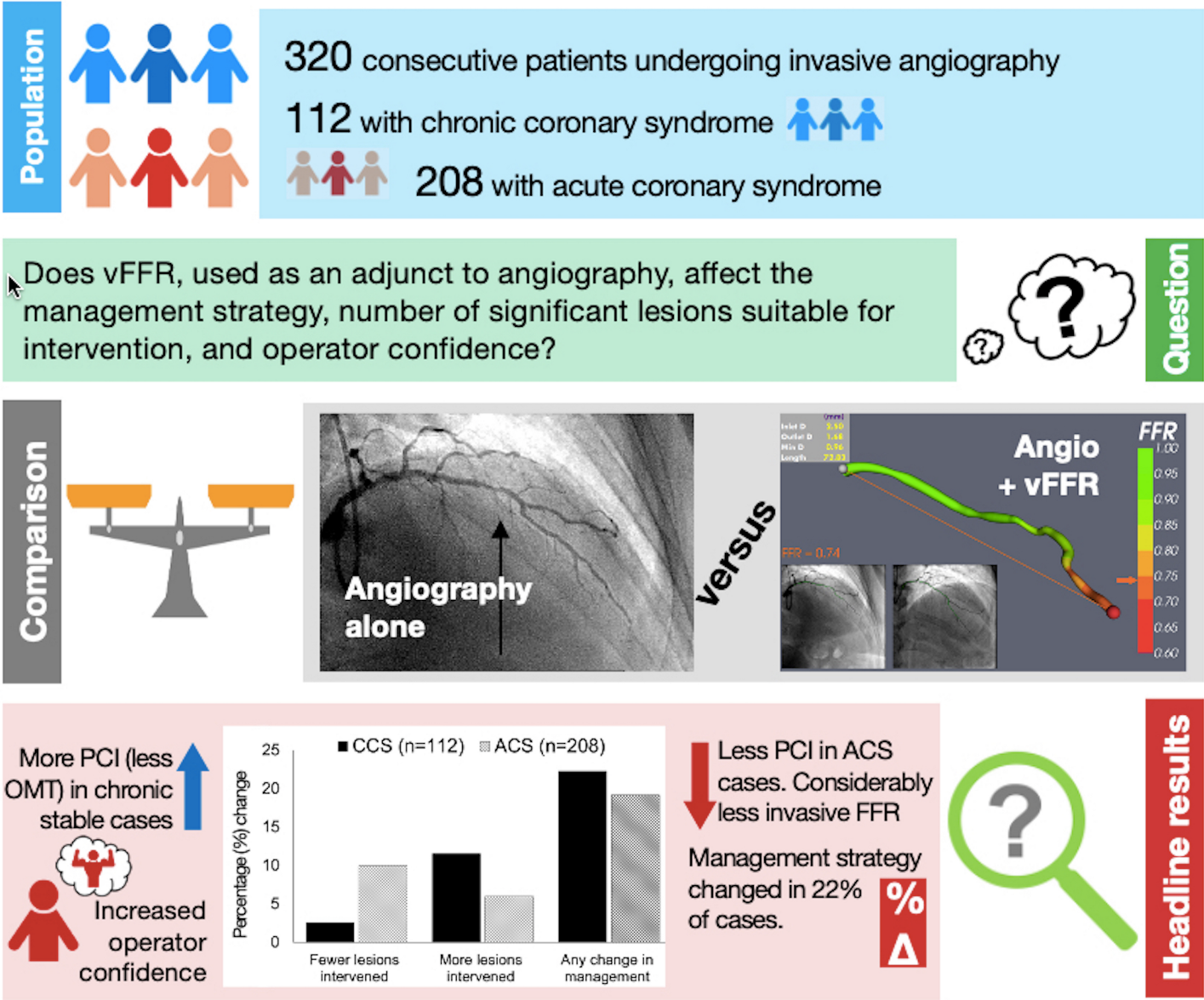

Supplement: Supplementary data [file heartjnl-2024-324039supp001.pdf]
